# Supplementary material for: Ecosystem services show variable responses to future climate conditions in the Colombian páramos
Source: PeerJ. 2021 May 3;9:e11370. doi: 10.7717/peerj.11370 (PMC8101452; doi:10.7717/peerj.11370)
Supplement: Supplemental Information 1 — The bootstrap resample is shown by dots for which the size is indicative of the number of replicates in the sample. The probability of being within the fundamental niche is calculated from Mahalanobis distances for each sample and shown as ellipsoids at the p>=0.9 (smallest ellipsoid), p>=0.5, and p>=0.1 (largest ellipsoid) thresholds for bootstrap resamples (A) 1, (B) 2, and (C) 1,000. [file peerj-09-11370-s001.pptx]

## Slide 1
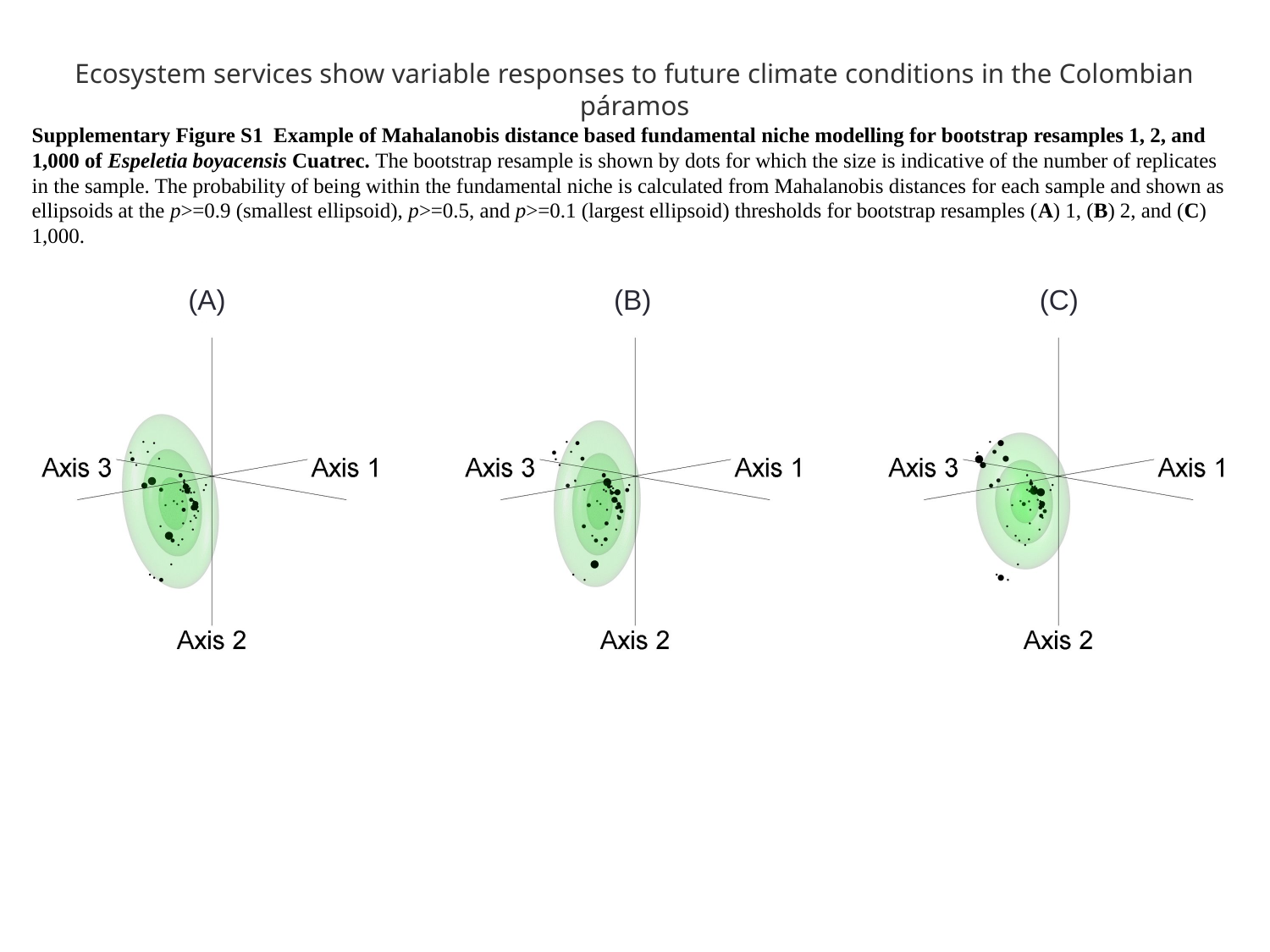

Ecosystem services show variable responses to future climate conditions in the Colombian páramos
Supplementary Figure S1  Example of Mahalanobis distance based fundamental niche modelling for bootstrap resamples 1, 2, and 1,000 of Espeletia boyacensis Cuatrec. The bootstrap resample is shown by dots for which the size is indicative of the number of replicates in the sample. The probability of being within the fundamental niche is calculated from Mahalanobis distances for each sample and shown as ellipsoids at the p>=0.9 (smallest ellipsoid), p>=0.5, and p>=0.1 (largest ellipsoid) thresholds for bootstrap resamples (A) 1, (B) 2, and (C) 1,000.
(A)
(C)
(B)
