# Supplementary material for: Ecosystem services show variable responses to future climate conditions in the Colombian páramos
Source: PeerJ. 2021 May 3;9:e11370. doi: 10.7717/peerj.11370 (PMC8101452; doi:10.7717/peerj.11370)
Supplement: Supplemental Information 2 — (A) A principal comment analysis (PCA) of the current climate conditions colour coded by axis dominance. The arrows show the direction and strength of the PCA loadings for the 19 bioclimatic variables. (B) It shows the same data in geographical space. [file peerj-09-11370-s002.pptx]

## Slide 1
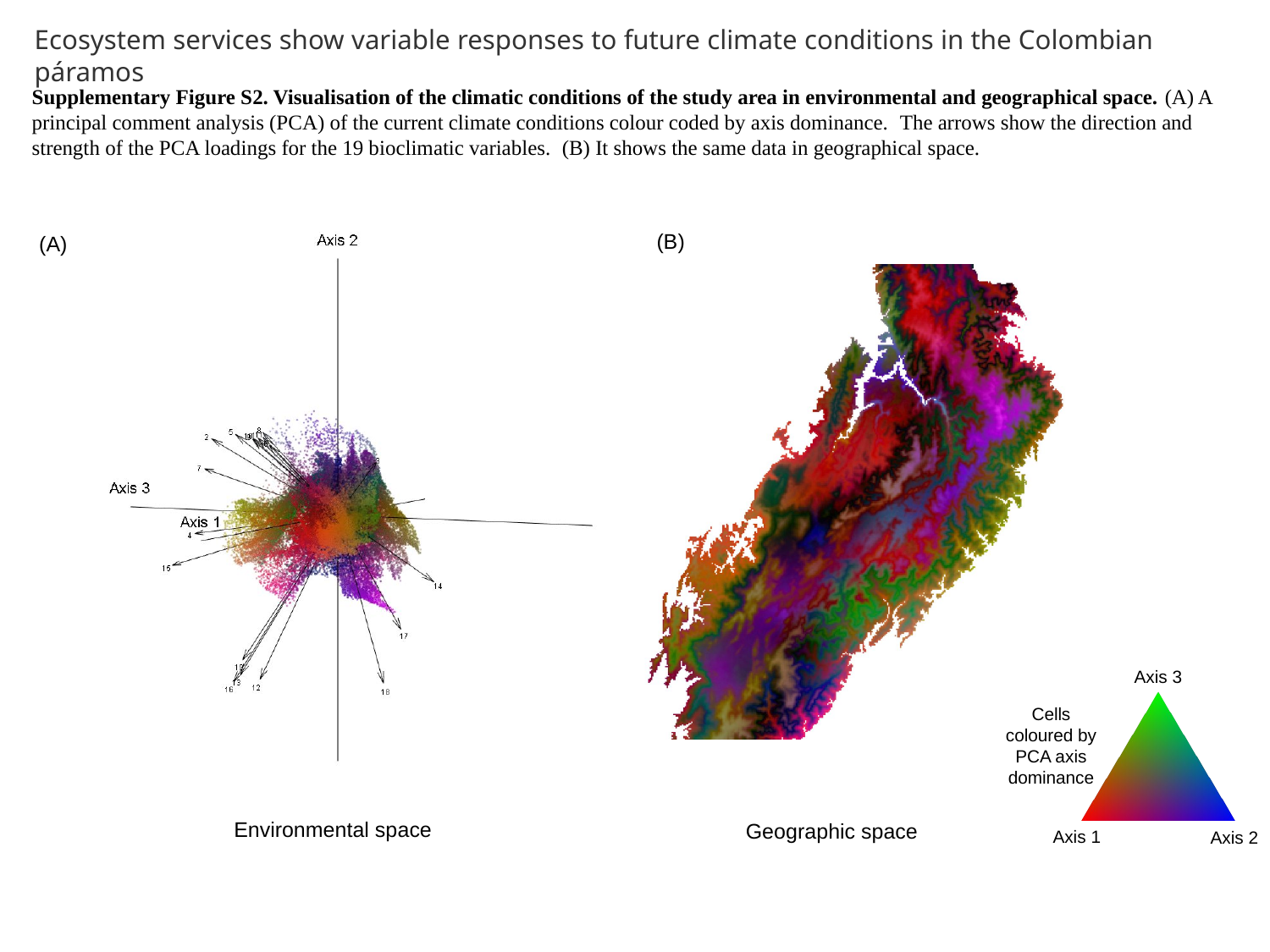

Ecosystem services show variable responses to future climate conditions in the Colombian páramos
Supplementary Figure S2. Visualisation of the climatic conditions of the study area in environmental and geographical space. (A) A principal comment analysis (PCA) of the current climate conditions colour coded by axis dominance. The arrows show the direction and strength of the PCA loadings for the 19 bioclimatic variables. (B) It shows the same data in geographical space.
(B)
(A)
Axis 3
Cells coloured by PCA axis dominance
Axis 1
Axis 2
Environmental space
Geographic space
