# Supplementary material for: Ecosystem services show variable responses to future climate conditions in the Colombian páramos
Source: PeerJ. 2021 May 3;9:e11370. doi: 10.7717/peerj.11370 (PMC8101452; doi:10.7717/peerj.11370)
Supplement: Supplemental Information 3 — A principal component analysis (PCA) was applied to present-day bioclimatic variables to create three-dimensional climate space. The core areas (p=0.75) of current and future climate are shown as ellipses. The current climate conditions are visualised by the black ellipse, with the 10 coloured ellipses visualising the core area of future climate conditions for each general circulation model (GCM) at 2070 under relative concentration pathway 8.5, which represents the most extreme future scenario. [file peerj-09-11370-s003.pptx]

## Slide 1
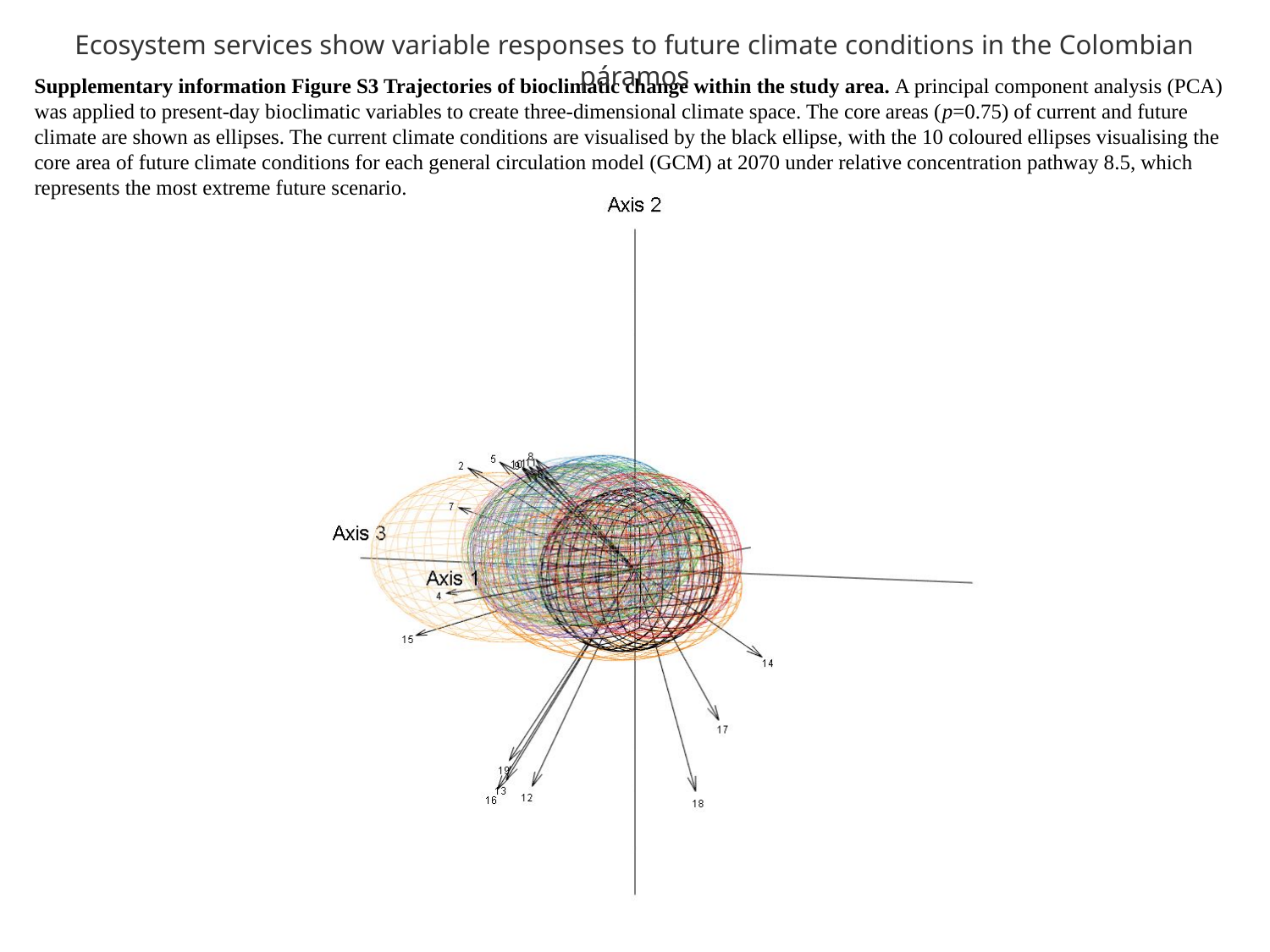

Ecosystem services show variable responses to future climate conditions in the Colombian páramos
Supplementary information Figure S3 Trajectories of bioclimatic change within the study area. A principal component analysis (PCA) was applied to present-day bioclimatic variables to create three-dimensional climate space. The core areas (p=0.75) of current and future climate are shown as ellipses. The current climate conditions are visualised by the black ellipse, with the 10 coloured ellipses visualising the core area of future climate conditions for each general circulation model (GCM) at 2070 under relative concentration pathway 8.5, which represents the most extreme future scenario.
