# Supplementary material for: Ecosystem services show variable responses to future climate conditions in the Colombian páramos
Source: PeerJ. 2021 May 3;9:e11370. doi: 10.7717/peerj.11370 (PMC8101452; doi:10.7717/peerj.11370)
Supplement: Supplemental Information 4 — Future scenario is for the year 2070 with RCP 8.5. Green ovals correspond to the core environmental niche of Espeletia boyacensis Cuatrec. [file peerj-09-11370-s004.pptx]

## Slide 1
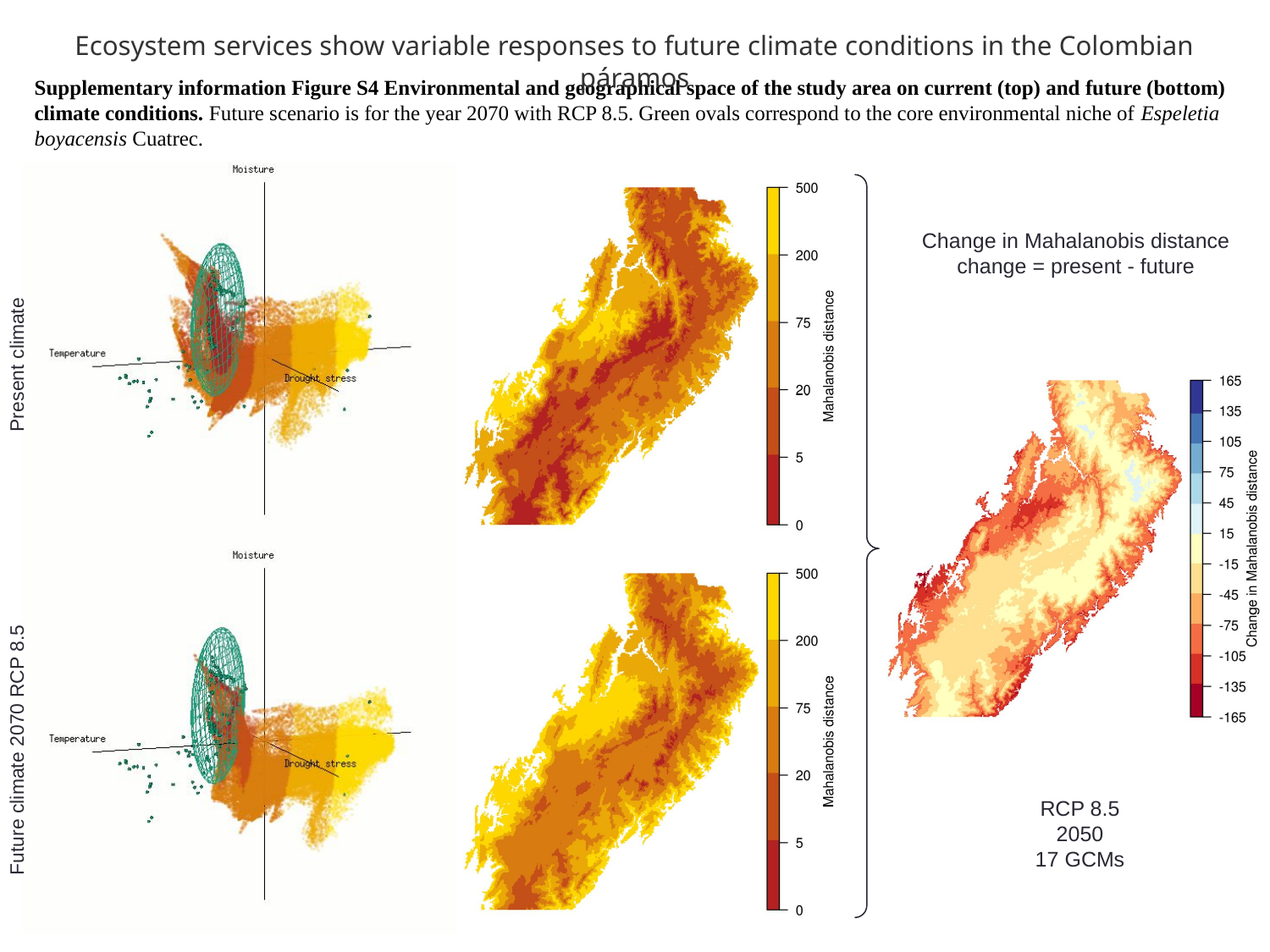

Ecosystem services show variable responses to future climate conditions in the Colombian páramos
Supplementary information Figure S4 Environmental and geographical space of the study area on current (top) and future (bottom) climate conditions. Future scenario is for the year 2070 with RCP 8.5. Green ovals correspond to the core environmental niche of Espeletia boyacensis Cuatrec.
Change in Mahalanobis distance
change = present - future
Present climate
Future climate 2070 RCP 8.5
RCP 8.5
2050
17 GCMs
