# Supplementary figures and images for: Ecosystem services show variable responses to future climate conditions in the Colombian páramos
Source: PeerJ. 2021 May 3;9:e11370. doi: 10.7717/peerj.11370 (PMC8101452; doi:10.7717/peerj.11370)

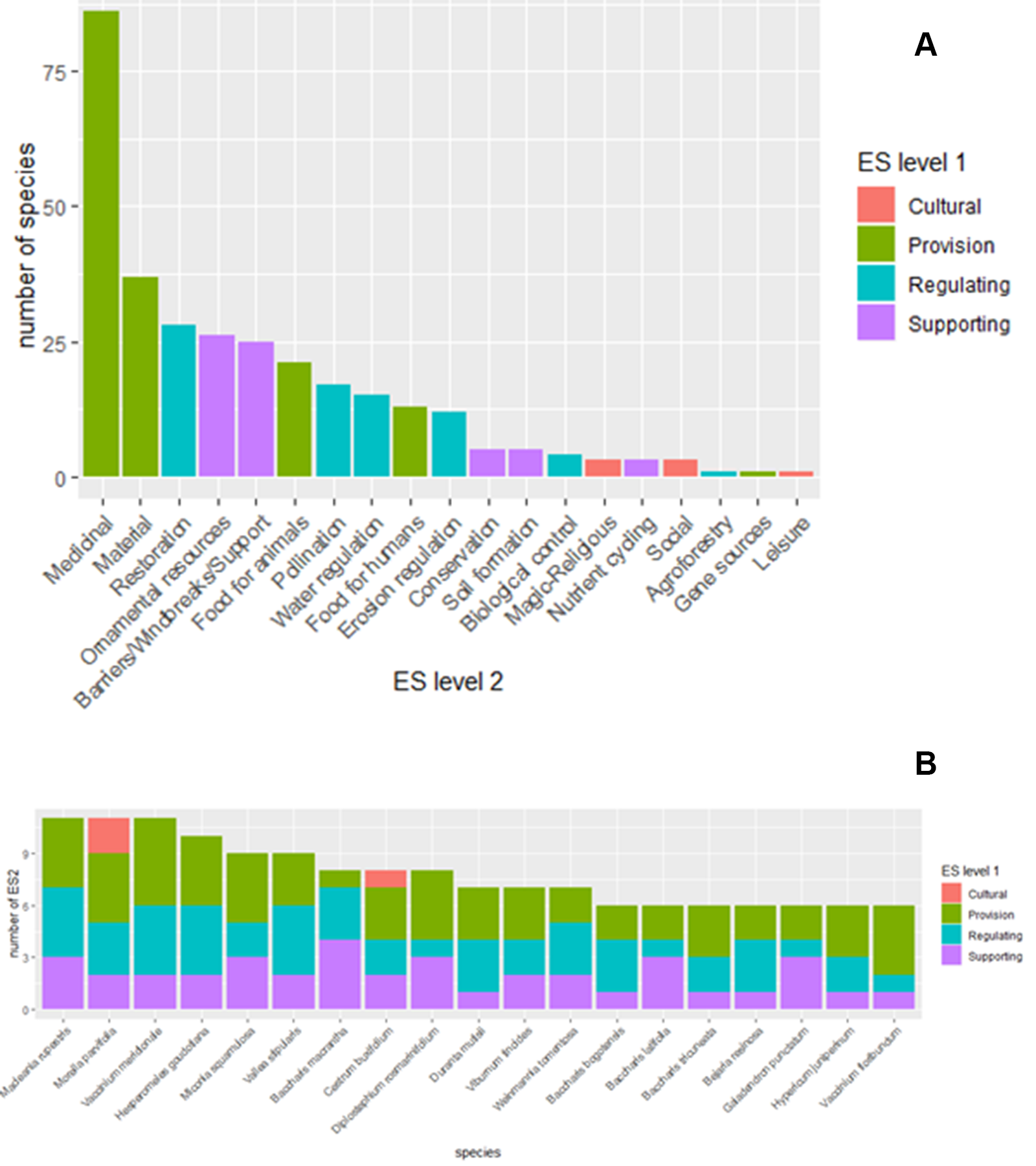

Supplement: Supplemental Information 5 — (a) Number of species by ecosystem services (at levels 1 and 2); (b) number of ecosystem services level 2 (ES2) per species, for species that provide more than five ES2. [file peerj-09-11370-s005.png]
