# Supplementary material for: Ecosystem services show variable responses to future climate conditions in the Colombian páramos
Source: PeerJ. 2021 May 3;9:e11370. doi: 10.7717/peerj.11370 (PMC8101452; doi:10.7717/peerj.11370)
Supplement: Supplemental Information 6 — Sources were searched using five keywords: páramos, ecosystem services, uses, traits and Boyacá. [file peerj-09-11370-s006.docx]

**Supplemental Table S1 – Literature review on useful plants from the páramos of Boyacá.** Sources were searched using five keywords: páramos, ecosystem services, uses, traits and Boyacá.

| **Year** | **Title** | **Type** | **Journal/Institution** | **Vol (date)** | **Issue** | **Pages** |
| --- | --- | --- | --- | --- | --- | --- |
| 2006 | Uso tradicional de plantas medicinales en la Vereda San Isidro, municipio de San José de Pare-Boyacá: un estudio preliminar usando técnicas cuantitativas | Scientific article | Acta Biológica Colombiana | 11 | 2 | 137-146 |
| 2007 | Caracterización fisicoquímica y organoléptica del fruto de agraz (*Vaccinium meridionale* Swartz) almacenado a 2ºC | Scientific article | Revista Facultad Nacional de Agronomía Medellín | 60 |  | 4179-4193 |
| 2007 | Estudio etnobotánico de especies vegetales con propiedades medicinales en seis municipios de Boyacá, Colombia | Scientific article | Actualidades biológicas | 29 |  | 87-96 |
| 2008 | Fibras vegetales utilizadas en artesanías en Colombia | Book | Artesanías de Colombia S.A., Instituto de Ciencias Naturales-Universidad Nacional de Colombia |  |  | 328 |
| 2009 | Especies útiles andinas | Book | Botanical Garden José Celestino Mutis-Bogotá | I |  | 242 |
| 2009 | Especies útiles andinas | Book | Botanical Garden José Celestino Mutis-Bogotá | II |  | 286 |
| 2009 | Perspectivas de cultivo de agraz o mortiño (*Vaccinium meridionale* Swartz) en la zona altoandina de Colombia | Book | Universidad Nacional de Colombia Facultad de Agronomía. Sede Bogotá |  |  | 140 |
| 2010 | 100 plantas útiles de Rabanal | Guide | Proyecto Páramo Andino (IAvH, CAR, CORPOBOYACA, CORPOCHIVOR) |  |  | 184 |
| 2010 | Caracterización y usos tradicionales de productos forestales no maderables (PFNM) en el corredor de conservación Guantiva - La Rusia - Iguaque | Scientific article | Colombia Forestal | 13 | 1 | 117-140 |
| 2011 | Conocimiento etnobotánico, patrones de uso y manejo de plantas útiles en la cuenca del Río Cane-Iguaque (Boyacá – Colombia); una aproximación desde los sistemas de uso de la biodiversidad | Scientific article | Ambiente & Sociedad | 14 | 1 | 45-75 |
| 2012 | Especies vegetales aromáticas de la provincia de Sumapaz y la cuenca del río Chicamocha en Colombia: Programa de aprovechamiento de aceites esenciales de especies nativas y foráneas promisorias para uso agrícola en Colombia | Book | Universidad Nacional de Colombia Facultad de Agronomía. Sede Bogotá |  |  | 189 |
| 2013 | Cariotipo de Espeletiopsis muiska | Scientific article | Revista MVZ Córdoba | 18 | 3 | 3868-3876 |
| 2013 | Use and valuation of native and introduced medicinal plant species in Campo Hermoso and Zetaquirá, Boyacá, Colombia | Scientific article | Journal of Ethnobiology and Ethnomedicine | 9 | 1 | 1-14 |
| 2014 | 2014 Cabrera et al. Restauración ecológica de los páramos de Colombia: Transformación y herramientas para su conservación | Book | Instituto de Investigación de Recursos Biológicos Alexander von Humboldt (IAvH) |  |  | 296 |
| 2014 | Crecimiento de *Baccharis macrantha* y *Viburnum triphyllum*, dos especies nativas útiles en restauración ecológica, plantadas en un pastizal andino (Boyacá, Colombia) | Scientific article | Biota Colombiana | 15 | 2 | 27-38 |
| 2014 | Especies forestales representativas del suroriente de Boyacá. Árboles de CORPOCHIVOR | Book | Corporación Autónoma Regional de Chivor – CORPOCHIVOR, Universidad Distrital Francisco José de Caldas. |  |  | 273 |
| 2014 | Etnobotánica y usos de las plantas de la comunidad rural del municipio de Cerinza, Boyacá, Colombia | Scientific article | Prospectiva Científica |  | 10 | 38-54 |
| 2015 | Plantas del páramo y sus usos para el buen vivir: páramos de Guerrero y Rabanal | Book | Instituto de Investigación de Recursos Biológicos Alexander von Humboldt (IAvH) | 2 |  | 60 |
| 2016 | Fichas técnicas de frutos silvestres en zonas andinas: Agraz | Technical Report-NGO | AgroDiva Foundation | 20/05/2016 |  | 1-9 |
| 2016 | Fichas técnicas de frutos silvestres en zonas andinas: Uva camarona | Technical Report-NGO | AgroDiva Foundation | 21/06/2016 |  | 1-7 |
| 2016 | Fichas técnicas de frutos silvestres en zonas andinas: Uva de anís | Technical Report-NGO | AgroDiva Foundation | 27/09/2016 |  | 1-7 |
| 2016 | Productos Forestales no maderables de CORPOCHIVOR: Una mirada a los regalos del bosque | Book | Corporación Autónoma Regional de Chivor – CORPOCHIVOR, Universidad Distrital Francisco José de Caldas. |  |  | 282 |
| 2017 | Descripción del uso y manejo de plantas medicinales de expendios de plaza de mercado de Tunja – Colombia | Poster | IX-Congreso Colombiano de Botánica |  |  |  |
| 2017 | Fichas técnicas de frutos silvestres en zonas andinas: Agraz-updated description | Technical Report-NGO | AgroDiva Foundation | 25/01/2017 |  | 1-6 |
| 2017 | Fichas técnicas de frutos silvestres en zonas andinas: Tuno Esmeraldo | Technical Report-NGO | AgroDiva Foundation | 25/01/2017 |  | 1-6 |
| 2018 | Useful plants of Boyacá Database (http://upb.plantsoftheworld.online/) | Online database | Royal Botanic Gardens, Kew (online resource: https://www.kew.org/science/our-science/projects/boyaca-useful-plants) | 01/11/2018 |  |  |
