# Supplementary material for: Ecosystem services show variable responses to future climate conditions in the Colombian páramos
Source: PeerJ. 2021 May 3;9:e11370. doi: 10.7717/peerj.11370 (PMC8101452; doi:10.7717/peerj.11370)
Supplement: Supplemental Information 7 — Only levels 1 (upper case) and 2 (lower case) are displayed. [file peerj-09-11370-s007.docx]

**Supplemental Table S2 – Classification of plant uses according to Cook (1995).** Only levels 1 (upper case) and 2 (lower case) are displayed.

| **ID_Number** | **LEVEL 1 - Level 2** |
| --- | --- |
| 100 | FOOD |
| 101 | FOOD - Unspecified Parts |
| 102 | FOOD - Entire Plant |
| 103 | FOOD - Unspecified Aerial Parts |
| 104 | FOOD - Seedlings/Germinated Seeds |
| 105 | FOOD - Galls |
| 106 | FOOD - Stems |
| 107 | FOOD - Bark |
| 108 | FOOD - Leaves |
| 109 | FOOD - Inflorescences |
| 110 | FOOD - Infructescences |
| 111 | FOOD - Seeds |
| 112 | FOOD - 'Roots' |
| 113 | FOOD - Exudates |
| 200 | FOOD ADDITIVES |
| 201 | FOOD ADDITIVES - Unspecified Parts |
| 202 | FOOD ADDITIVES - Entire Plant |
| 203 | FOOD ADDITIVES - Unspecified Aerial Parts |
| 204 | FOOD ADDITIVES - Seedlings/Germinated Seeds |
| 205 | FOOD ADDITIVES - Galls |
| 206 | FOOD ADDITIVES - Stems |
| 207 | FOOD ADDITIVES - Bark |
| 208 | FOOD ADDITIVES - Leaves |
| 209 | FOOD ADDITIVES - Inflorescences |
| 210 | FOOD ADDITIVES - Infructescences |
| 211 | FOOD ADDITIVES - Seeds |
| 212 | FOOD ADDITIVES - 'Roots' |
| 213 | FOOD ADDITIVES - Exudates |
| 300 | ANIMAL FOOD |
| 301 | ANIMAL FOOD - Unspecified Parts |
| 302 | ANIMAL FOOD - Bark |
| 303 | ANIMAL FOOD - 'Roots' |
| 304 | ANIMAL FOOD - Exudates |
| 305 | ANIMAL FOOD - Fertile Plant Parts |
| 306 | ANIMAL FOOD - Aerial Parts |
| 307 | ANIMAL FOOD - Other Parts |
| 400 | BEE PLANTS |
| 500 | INVERTEBRATE FOOD |
| 600 | MATERIALS |
| 601 | MATERIALS - Unspecified Materials |
| 602 | MATERIALS - Fibres |
| 603 | MATERIALS - Cane etc. |
| 604 | MATERIALS - Wood |
| 605 | MATERIALS - Cork/Cork Substitutes |
| 606 | MATERIALS - Gums/Resins |
| 607 | MATERIALS - Latex/Rubber |
| 608 | MATERIALS - Tannins/Dyestuffs |
| 609 | MATERIALS - Lipids |
| 610 | MATERIALS - Essential Oils |
| 611 | MATERIALS - Waxes |
| 612 | MATERIALS - Alcohols |
| 613 | MATERIALS - Other Materials/Chemicals |
| 700 | FUELS |
| 701 | FUELS - Unspecified Fuels |
| 702 | FUELS - Miscellaneous Fuels |
| 703 | FUELS - Fuelwood |
| 704 | FUELS - Charcoal |
| 705 | FUELS - Petroleum Substitutes, Alcohols etc. |
| 706 | FUELS - Tinder |
| 800 | SOCIAL USES |
| 801 | SOCIAL USES - Unspecified Social Uses |
| 802 | SOCIAL USES - Smoking Materials/Drugs |
| 803 | SOCIAL USES - Antifertility Agents |
| 804 | SOCIAL USES - 'Religious' Uses |
| 805 | SOCIAL USES - Miscellaneous Social Uses |
| 900 | VERTEBRATE POISONS |
| 901 | VERTEBRATE POISONS - Unspecified Vertebrates |
| 902 | VERTEBRATE POISONS - Fish |
| 903 | VERTEBRATE POISONS - Amphibians |
| 904 | VERTEBRATE POISONS - Reptiles |
| 905 | VERTEBRATE POISONS - Birds |
| 906 | VERTEBRATE POISONS - Mammals |
| 1000 | NON-VERTEBRATE POISONS |
| 1001 | NON-VERTEBRATE POISONS - Unspecified Non-Vertebrates |
| 1002 | NON-VERTEBRATE POISONS - Unspecified Microbes |
| 1003 | NON-VERTEBRATE POISONS - Viruses |
| 1004 | NON-VERTEBRATE POISONS - Bacteria |
| 1005 | NON-VERTEBRATE POISONS - Fungi |
| 1006 | NON-VERTEBRATE POISONS - Plants |
| 1007 | NON-VERTEBRATE POISONS - Protozoa |
| 1008 | NON-VERTEBRATE POISONS - Mollusca |
| 1009 | NON-VERTEBRATE POISONS - Arthropoda |
| 1010 | NON-VERTEBRATE POISONS - Other Eumetazoa |
| 1100 | MEDICINES |
| 1101 | MEDICINES - Unspecified Medicinal Disorders |
| 1102 | MEDICINES - Abnormalities |
| 1103 | MEDICINES - Blood System Disorders |
| 1104 | MEDICINES - Circulatory System Disorders |
| 1105 | MEDICINES - Digestive System Disorders |
| 1106 | MEDICINES - Endocrine System Disorders |
| 1107 | MEDICINES - Genitourinary System Disorders |
| 1108 | MEDICINES - Ill-defined Symptoms |
| 1109 | MEDICINES - Immune System Disorders |
| 1110 | MEDICINES - Infections/Infestations |
| 1111 | MEDICINES - Inflammation |
| 1112 | MEDICINES - Injuries |
| 1113 | MEDICINES - Mental Disorders |
| 1114 | MEDICINES - Metabolic System Disorders |
| 1115 | MEDICINES - Muscular-Skeletal System Disorders |
| 1116 | MEDICINES - Neoplasms |
| 1117 | MEDICINES - Nervous System Disorders |
| 1118 | MEDICINES - Nutritional Disorders |
| 1119 | MEDICINES - Pain |
| 1120 | MEDICINES - Poisonings |
| 1121 | MEDICINES - Pregnancy/Birth/Puerpuerium Disorders |
| 1122 | MEDICINES - Respiratory System Disorders |
| 1123 | MEDICINES - Sensory System Disorders |
| 1124 | MEDICINES - Skin/Subcutaneous Cellular Tissue Disorders |
| 1200 | ENVIRONMENTAL USES |
| 1201 | ENVIRONMENTAL USES - Unspecified Environmental Uses |
| 1202 | ENVIRONMENTAL USES - Erosion Control |
| 1203 | ENVIRONMENTAL USES - Shade/Shelter |
| 1204 | ENVIRONMENTAL USES - Revegetators |
| 1205 | ENVIRONMENTAL USES - Indicators |
| 1206 | ENVIRONMENTAL USES - Soil Improvers |
| 1207 | ENVIRONMENTAL USES - Ornamentals |
| 1208 | ENVIRONMENTAL USES - Boundaries/Barriers/Supports |
| 1209 | ENVIRONMENTAL USES - Agroforestry |
| 1210 | ENVIRONMENTAL USES - Firebreaks |
| 1211 | ENVIRONMENTAL USES - Pollution Control |
| 1300 | GENE SOURCES |
