# Supplementary material for: Ecosystem services show variable responses to future climate conditions in the Colombian páramos
Source: PeerJ. 2021 May 3;9:e11370. doi: 10.7717/peerj.11370 (PMC8101452; doi:10.7717/peerj.11370)
Supplement: Supplemental Information 8 — Species names and families are based on the taxonomic backbone of ColPlantA (http://colplanta.org/). Elevations and habits follow the Colombian Catalogue of Plants and Lichens (Bernal, Gradstein & Celis, 2019). [file peerj-09-11370-s008.docx]

**Supplemental Table S3 – Selected plant species.** Species names and families are based on the taxonomic backbone of Col*Plant*A (<http://colplanta.org/>). Elevations and habits follow the Colombian Catalogue of Plants and Lichens (Bernal, Gradstein & Celis, 2019).

| **No** | **Species name** | **Family** | **Habit** | **Andean forest** | **Low páramo**  **(or sub-páramo)** | **Mid-paramo and**  **super-páramo** | **Elevation range (m a.s.l.)** | **Number of records** | **CULTURAL** | | | **PROVISION** | | | | | **REGULATING** | | | | | | **SUPPORTING** | | | | |
| --- | --- | --- | --- | --- | --- | --- | --- | --- | --- | --- | --- | --- | --- | --- | --- | --- | --- | --- | --- | --- | --- | --- | --- | --- | --- | --- | --- |
|  |  |  |  |  |  |  |  |  | **Leisure** | **Magic-Religious** | **Social** | **Food for animals** | **Gene sources** | **Food for humans** | **Material** | **Medicinal** | **Agroforestry** | **Biological control** | **Erosion regulation** | **Pollination** | **Restoration** | **Water regulation** | **Barriers/Windbreaks/**  **Support** | **Conservation** | **Nutrient cycling** | **Ornamental resources** | **Soil formation** |
| 1 | *Acaena cylindristachya* | Rosaceae | Basal rosette | X | X | XX | 2500–4300 | 272 |  |  |  |  |  |  |  | x |  |  |  |  |  |  |  |  |  |  |  |
| 2 | *Acaena elongata* | Rosaceae | Prostrate shrub | X | XX | XX | 2500–4000 | 687 |  |  |  |  |  |  |  | x |  |  |  |  |  |  |  |  |  |  |  |
| 3 | *Arenaria lanuginosa* | Caryophyllaceae | Prostrate herb | X | XX | XX | 1200–3400 | 600 |  |  |  |  |  |  |  | x |  |  |  |  |  |  |  |  |  |  |  |
| 4 | *Baccharis bogotensis* | Asteraceae | Upright shrub | X | XX | X | 1950–4100 | 44 |  |  |  |  |  |  | x | x |  |  | x | x |  | x | x |  |  |  |  |
| 5 | *Baccharis latifolia* | Asteraceae | Upright shrub | X | XX | X | 1400–4000 | 722 |  |  |  |  |  |  | x | x |  |  |  |  | x |  | x |  |  | x | x |
| 6 | *Baccharis macrantha* | Asteraceae | Upright shrub | X | X | X | 1700–4100 | 165 |  |  |  |  |  |  | x |  |  |  | x | x | x |  | x |  | x | x | x |
| 7 | *Baccharis tricuneata* | Asteraceae | Upright shrub | X | XX | X | 1850–4400 | 468 |  |  |  | x |  |  | x | x |  | x |  |  | x |  | x |  |  |  |  |
| 8 | *Barnadesia spinosa* | Asteraceae | Upright shrub | XX | XX | X | 1950–4100 | 62 |  |  |  |  |  |  |  | x |  |  |  | x | x |  | x |  |  |  |  |
| 9 | *Bartsia santolinifolia* | Orobanchaceae | Upright herb | - | - | X | 2900–4400 | 288 |  |  |  |  |  |  |  | x |  |  |  |  |  |  |  |  |  |  |  |
| 10 | *Bejaria resinosa* | Ericaceae | Upright shrub | X | XX | X | 1750–3900 | 890 |  |  |  |  |  |  | x | x |  |  | x | x | x |  |  |  |  | x |  |
| 11 | *Berberis glauca* | Berberidaceae | Upright shrub | X | XX | - | 2400–3200 | 24 |  |  |  |  |  |  |  | x |  |  |  |  |  |  |  |  |  |  |  |
| 12 | *Berberis rigidifolia* | Berberidaceae | Upright shrub | X | XX | X | 1750–4200 | 49 |  |  |  |  |  |  |  | x |  |  |  |  |  |  | x |  |  |  |  |
| 13 | *Brachyotum strigosum* | Melastomataceae | Upright shrub | X | XX | X | 2500–3900 | 418 |  |  |  |  |  |  |  | x |  |  |  |  | x |  | x |  |  | x |  |
| 14 | *Bucquetia glutinosa* | Melastomataceae | Upright shrub | X | XX | X | 2100–4104 | 1271 |  |  |  | x |  |  | x |  |  |  |  |  | x |  |  |  |  |  |  |
| 15 | *Calamagrostis effusa* | Poaceae | Tussock | - | X | XX | 2500–4500 | 2215 |  |  |  |  |  |  | x | x |  |  | x |  |  | x |  |  |  |  |  |
| 16 | *Carex pichinchensis* | Cyperaceae | Tussock | X | X | X | 1325–4500 | 439 |  |  |  |  |  |  |  |  |  |  |  |  | x | x |  |  |  |  |  |
| 17 | *Castilleja fissifolia* | Orobanchaceae | Upright herb | X | X | XX | 2225–4300 | 898 |  |  |  | x |  |  |  |  |  |  |  |  |  |  |  |  |  | x |  |
| 18 | *Castratella piloselloides* | Melastomataceae | Basal rosette | X | X | XX | 2120–4020 | 481 |  |  |  |  |  |  |  | x |  |  |  |  |  |  |  |  |  |  |  |
| 19 | *Cavendishia bracteata* | Ericaceae | Upright shrub | X | XX | X | 1000–3820 | 336 |  |  |  | x |  | x | x | x |  |  |  |  | x |  |  |  |  |  |  |
| 20 | *Cerastium arvense* | Caryophyllaceae | Upright herb | X | X | XX | 2500–4900 | 335 |  |  |  |  |  |  |  | x |  |  |  |  |  |  |  |  |  |  |  |
| 21 | *Cestrum buxifolium* | Solanaceae | Upright shrub | X | XX | X | 1870–4100 | 134 |  | x |  | x |  |  | x | x |  |  |  | x | x |  | x |  |  | x |  |
| 22 | *Chaetolepis microphylla* | Melastomataceae | Upright herb | X | XX | X | 2000–3700 | 109 |  |  |  |  |  |  |  | x |  |  |  |  |  |  |  |  |  |  |  |
| 23 | *Chusquea tessellata* | Poaceae | Tussock | - | X | XX | 2800–4350 | 1472 |  |  |  |  |  |  | x |  |  |  |  |  | x |  |  |  |  |  |  |
| 24 | *Clethra fimbriata* | Clethraceae | Upright shrub | X | XX | X | 1830–3700 | 784 |  |  |  | x |  |  | x | x |  |  |  |  |  | x | x |  |  |  |  |
| 25 | *Culcitium canescens* | Asteraceae | Basal rosette | - | - | XX | 3100–4710 | 99 |  |  |  |  |  |  |  | x |  |  |  |  |  |  |  |  |  |  |  |
| 26 | *Cuphea dipetala* | Lythraceae | Upright herb | X | XX | - | 1400–3215 | 34 |  |  |  |  |  |  |  | x |  |  |  |  |  |  |  |  |  |  |  |
| 27 | *Diplostephium rosmarinifolium* | Asteraceae | Upright shrub | X | XX | XX | 2000–3900 | 528 |  |  |  | x |  | x | x | x |  |  |  |  | x |  | x | x |  | x |  |
| 28 | *Dodonaea viscosa* | Sapindaceae | Upright shrub | X | XX | X | 250–3900 | 21 |  |  |  |  |  |  | x | x |  |  |  |  |  |  |  |  |  |  |  |
| 29 | *Drimys granadensis* | Winteraceae | Tree | X | XX | X | 1800–3900 | 495 |  | x |  |  |  | x | x | x |  |  |  |  |  |  |  |  |  | x |  |
| 30 | *Dryopteris wallichiana* | Dryopteridaceae | Upright herb | XX | XX | X | 350–3600 | 210 |  |  |  |  |  |  |  | x |  |  |  |  |  |  |  |  |  | x |  |
| 31 | *Duranta mutisii* | Verbenaceae | Upright shrub | X | XX | X | 1300–3500 | 92 |  |  |  | x |  |  | x | x |  |  | x | x | x |  | x |  |  |  |  |
| 32 | *Eccremis coarctata* | Xanthorrhoeaceae | Upright herb | X | XX | X | 1990–3600 | 154 |  |  |  |  |  |  | x |  |  |  |  |  |  |  |  |  |  |  |  |
| 33 | *Echeveria quitensis* | Crassulaceae | Basal rosette | - | X | XX | 2700–4100 | 116 |  |  |  |  |  |  |  | x |  |  |  |  |  |  |  |  |  |  |  |
| 34 | *Elaphoglossum gayanum* | Dryopteridaceae | Upright herb | X | X | XX | 1700–4300 | 238 |  |  |  |  |  |  |  | x |  |  |  |  |  |  |  |  |  |  |  |
| 35 | *Equisetum bogotense* | Equisetaceae | Upright herb | X | X | XX | 1500–3800 | 495 |  |  |  |  |  |  |  | x |  |  |  |  |  |  |  |  |  |  |  |
| 36 | *Eryngium humile* | Apiaceae | Basal rosette | X | XX | XX | 2400–4500 | 623 |  |  |  |  |  |  |  | x |  |  |  |  |  |  |  |  |  |  |  |
| 37 | *Escallonia myrtilloides* | Escalloniaceae | Tree | X | XX | XX | 2500–3900 | 1183 |  |  |  |  |  |  |  | x |  |  |  |  |  | x |  |  |  |  |  |
| 38 | *Escallonia paniculata* | Escalloniaceae | Tree | X | XX | XX | 1500–3800 | 373 |  |  |  |  |  |  | x |  |  |  |  |  | x | x | x |  |  |  |  |
| 39 | *Espeletia arbelaezii* | Asteraceae | Stem rosette | - | XX | - | 3000–3300 | 31 |  |  | x |  |  |  |  |  |  |  |  |  |  |  |  | x |  |  |  |
| 40 | *Espeletia boyacensis* | Asteraceae | Basal rosette | - | XX | XX | 2345–3900 | 100 |  |  |  |  |  |  |  | x |  |  |  |  |  |  |  |  |  |  |  |
| 41 | *Espeletia congestiflora* | Asteraceae | Basal rosette | - | XX | XX | 2500–4000 | 73 |  |  |  |  |  |  |  | x |  |  |  |  |  |  |  |  |  |  |  |
| 42 | *Espeletia incana* | Asteraceae | Stem rosette | - | XX | XX | 2900–3900 | 27 |  |  |  |  |  |  | x | x |  |  |  |  |  |  |  |  |  | x |  |
| 43 | *Espeletia lopezii* | Asteraceae | Stem rosette | - | XX | XX | 3100–4550 | 80 |  |  |  |  |  |  |  | x |  |  |  |  |  |  |  |  |  | x |  |
| 44 | *Espeletia murilloi* | Asteraceae | Stem rosette | - | XX | XX | 2700–3700 | 76 |  |  |  |  |  |  |  |  |  |  |  |  |  |  |  | x |  |  |  |
| 45 | *Espeletiopsis guacharaca* | Asteraceae | Stem rosette | - | XX | XX | 2900–3700 | 30 |  |  |  |  |  |  |  | x |  |  |  |  |  |  |  |  |  | x |  |
| 46 | *Espeletiopsis pleiochasia* | Asteraceae | Stem rosette | X | XX | - | 2200–3624 | 43 |  |  |  |  |  |  |  | x |  |  |  |  |  |  |  |  |  |  |  |
| 47 | *Gaiadendron punctatum* | Loranthaceae | Upright shrub | X | XX | X | 1330–3950 | 2667 |  |  |  | x |  |  | x |  |  |  |  | x |  |  | x | x |  | x |  |
| 48 | *Galium hypocarpium* | Rubiaceae | Trailing herb | X | XX | XX | 700–4350 | 499 |  |  |  |  |  |  | x | x |  |  |  |  |  |  |  |  |  |  |  |
| 49 | *Gaultheria anastomosans* | Ericaceae | Prostrate shrub | X | XX | XX | 2500–4100 | 2275 |  |  |  | x |  |  |  | x |  |  |  |  | x |  | x |  | x |  |  |
| 50 | *Gaylussacia buxifolia* | Ericaceae | Upright shrub | X | XX | X | 1990–3500 | 559 |  |  |  |  |  | x |  |  |  |  |  |  |  |  |  |  |  |  |  |
| 51 | *Halenia asclepiadea* | Gentianaceae | Upright herb | - | X | XX | 2700–4000 | 427 |  |  |  |  |  |  |  | x |  |  |  |  |  |  |  |  |  |  |  |
| 52 | *Hesperomeles goudotiana* | Rosaceae | Upright shrub | - | XX | X | 2600–3700 | 263 |  |  |  | x |  | x | x | x |  |  | x | x | x | x | x |  |  | x |  |
| 53 | *Hieracium avilae* | Asteraceae | Upright herb | X | XX | XX | 2700–4350 | 379 |  |  |  |  |  |  |  | x |  |  |  |  |  |  |  |  |  |  |  |
| 54 | *Hypericum juniperinum* | Hypericaceae | Upright shrub | X | XX | X | 1990–3810 | 1534 |  |  |  | x |  |  | x | x |  |  |  |  | x | x | x |  |  |  |  |
| 55 | *Hypericum mexicanum* | Hypericaceae | Upright shrub | X | XX | XX | 1700–4200 | 364 |  |  |  |  |  |  |  | x |  |  |  |  |  |  |  |  |  |  |  |
| 56 | *Hypochaeris sessiliflora* | Asteraceae | Basal rosette | X | XX | XX | 2100–4900 | 496 |  |  |  |  |  |  |  | x |  |  |  |  |  |  |  |  |  |  |  |
| 57 | *Jamesonia bogotensis* | Pteridaceae | Upright herb | - | X | XX | 2950–4400 | 153 |  |  |  |  |  |  | x |  |  |  |  |  |  |  |  | x |  |  |  |
| 58 | *Juncus effusus* | Juncaceae | Upright herb | X | XX | XX | 1500–3700 | 3130 |  |  |  |  |  |  | x | x |  |  |  |  |  |  |  |  |  |  |  |
| 59 | *Lachemilla orbiculata* | Rosaceae | Cushion/mat forming | X | XX | XX | 1500–4000 | 538 |  |  |  |  |  |  |  | x |  |  |  |  |  |  |  |  |  |  |  |
| 60 | *Lobelia tenera* | Campanulaceae | Upright herb | X | XX | XX | 1830–3750 | 226 |  |  |  |  |  |  |  | x |  |  |  |  |  |  |  |  |  |  |  |
| 61 | *Lupinus alopecuroides* | Fabaceae | Upright herb | - | X | XX | 2700–4500 | 50 |  |  |  | x |  |  |  | x |  |  |  | x |  |  |  |  |  |  |  |
| 62 | *Lycopodium clavatum* | Lycopodiaceae | Prostrate herb | X | XX | XX | 1050–4200 | 88 |  |  |  |  |  |  |  | x |  |  |  |  |  |  |  |  |  |  |  |
| 63 | *Macleania rupestris* | Ericaceae | Upright shrub | X | XX | X | 2000–4100 | 497 |  |  |  | x |  | x | x | x | x |  | x | x | x |  | x |  |  | x | x |
| 64 | *Margyricarpus pinnatus* | Rosaceae | Prostrate shrub | X | XX | X | 2500–3600 | 193 |  |  |  |  |  |  |  | x |  |  |  |  |  |  |  |  |  |  |  |
| 65 | *Miconia squamulosa* | Melastomataceae | Upright shrub | XX | XX | X | 2100–3600 | 279 |  |  |  | x |  | x | x | x |  |  |  | x | x |  | x |  |  | x | x |
| 66 | *Monnina aestuans* | Polygalaceae | Upright shrub | X | XX | XX | 1700–4500 | 272 |  |  |  |  |  |  |  | x |  |  |  |  | x |  |  |  |  |  |  |
| 67 | *Monnina salicifolia* | Polygalaceae | Upright shrub | X | XX | XX | 1900–4150 | 423 |  |  |  |  |  |  | x | x |  |  |  |  |  |  | x |  |  |  |  |
| 68 | *Morella parvifolia* | Myricaceae | Upright shrub | X | XX | XX | 1600–3800 | 442 |  | x | x | x |  | x | x | x |  |  | x |  | x | x | x |  |  | x |  |
| 69 | *Mutisia clematis* | Asteraceae | Trailing herb | X | XX | X | 1990–3900 | 69 |  |  | x |  |  |  | x |  |  |  |  |  |  |  |  |  |  |  |  |
| 70 | *Myrsine coriacea* | Primulaceae | Upright shrub | XX | XX | X | 820–3360 | 498 |  |  |  | x |  |  | x | x |  |  |  |  |  |  |  |  |  |  |  |
| 71 | *Nertera granadensis* | Rubiaceae | Prostrate herb | X | XX | XX | 1300–4300 | 190 |  |  |  |  |  | x |  | x |  |  | x |  |  | x |  |  |  | x |  |
| 72 | *Noticastrum marginatum* | Asteraceae | Upright herb | X | XX | XX | 1900–4200 | 180 |  |  |  |  |  |  |  | x |  |  |  |  |  |  |  |  |  |  |  |
| 73 | *Oreopanax mutisianus* | Araliaceae | Tree | X | XX | X | 2700–3700 | 126 |  |  |  |  |  |  |  | x |  |  |  |  |  |  |  |  |  |  |  |
| 74 | *Orthrosanthus chimboracensis* | Iridaceae | Basal rosette | X | XX | XX | 2200–4000 | 1007 |  |  |  |  |  |  | x |  |  |  |  |  |  |  |  |  |  | x |  |
| 75 | *Oxalis medicaginea* | Oxalidaceae | Upright herb | X | XX | XX | 1400–4000 | 123 |  |  |  |  |  |  |  | x |  |  |  |  |  |  |  |  |  |  |  |
| 76 | *Paepalanthus dendroides* | Eriocaulaceae | Basal rosette | X | XX | X | 2400–3575 | 82 |  |  |  |  |  |  |  |  |  |  |  |  |  | x |  |  |  |  | x |
| 77 | *Paramiflos glandulosus* | Asteraceae | Basal rosette | - | XX | XX | 1990–3650 | 48 |  |  |  |  |  |  |  | x |  |  |  |  |  |  |  |  |  | x |  |
| 78 | *Pentacalia abietina* | Asteraceae | Upright shrub | X | XX | XX | 1750–4200 | 405 |  |  |  |  |  |  |  | x |  |  |  |  |  |  |  |  |  |  |  |
| 79 | *Pentacalia andicola* | Asteraceae | Upright shrub | X | XX | XX | 2400–4630 | 488 |  |  |  |  |  |  |  | x |  |  |  |  |  |  |  |  |  |  |  |
| 80 | *Pentacalia corymbosa* | Asteraceae | Upright shrub | X | XX | XX | 1800–4000 | 65 |  |  |  |  |  |  |  | x |  |  |  |  |  |  |  |  |  |  |  |
| 81 | *Peperomia hartwegiana* | Piperaceae | Prostrate herb | X | XX | XX | 1400–4000 | 237 |  |  |  |  |  |  |  | x |  |  |  |  |  |  |  |  |  |  |  |
| 82 | *Pernettya prostrata* | Ericaceae | Prostrate herb | X | XX | XX | 2000–4525 | 4748 |  |  |  |  |  |  |  | x |  | x |  |  |  |  |  |  |  |  |  |
| 83 | *Phytolacca bogotensis* | Phytolaccaceae | Upright herb | X | XX | - | 1560–3600 | 282 |  |  |  |  |  |  |  | x |  | x |  |  |  |  |  |  |  |  |  |
| 84 | *Polylepis quadrijuga* | Rosaceae | Tree | - | XX | X | 2800–4000 | 56 |  |  |  |  |  |  | x |  |  |  |  |  |  |  | x |  |  |  |  |
| 85 | *Puya goudotiana* | Bromeliaceae | Basal rosette | - | X | XX | 2760–3550 | 220 |  |  |  |  |  |  |  |  |  |  | x | x | x | x | x |  |  |  |  |
| 86 | *Ribes andicola* | Grossulariaceae | Upright shrub | X | XX | X | 2800–3720 | 90 |  |  |  |  |  |  |  | x |  |  |  |  |  |  |  |  |  |  |  |
| 87 | *Rubus acanthophyllos* | Rosaceae | Prostrate herb | X | XX | XX | 2800–4000 | 314 |  |  |  |  |  |  |  | x |  |  |  |  |  |  |  |  |  |  |  |
| 88 | *Salvia amethystina* | Lamiaceae | Upright herb | X | XX | XX | 2300–4000 | 55 |  |  |  |  |  |  |  | x |  |  |  |  |  |  |  |  |  |  |  |
| 89 | *Senecio formosoides* | Asteraceae | Upright herb | - | X | XX | 3000–4425 | 74 |  |  |  |  |  |  |  | x |  | x |  |  |  |  |  |  |  |  |  |
| 90 | *Senecio niveoaureus* | Asteraceae | Basal rosette | - | X | XX | 3000–4600 | 74 |  |  |  |  |  |  |  | x |  |  |  | x |  |  |  |  |  | x |  |
| 91 | *Siphocampylus columnae* | Campanulaceae | Upright shrub | - | XX | XX | 2100–4100 | 351 |  |  |  |  |  |  |  | x |  |  |  |  |  |  |  |  |  |  |  |
| 92 | *Stevia lucida* | Asteraceae | Upright shrub | X | XX | XX | 250–4900 | 438 |  |  |  |  |  |  |  | x |  |  |  |  |  |  |  |  |  |  |  |
| 93 | *Symplocos theiformis* | Symplocaceae | Upright shrub | - | XX | X | 2450–3724 | 492 | x |  |  |  |  | x |  | x |  |  |  |  | x |  |  |  |  |  |  |
| 94 | *Ugni myricoides* | Myrtaceae | Upright shrub | X | XX | X | 2190–3500 | 717 |  |  |  |  |  |  |  | x |  |  |  |  |  |  |  |  |  |  |  |
| 95 | *Ullucus tuberosus* | Basellaceae | Upright herb | XX | XX | - | 2250–3400 | 474 |  |  |  |  |  | x |  | x |  |  |  |  |  |  |  |  |  |  |  |
| 96 | *Uncinia hamata* | Cyperaceae | Tussock | XX | XX | X | 910–3650 | 653 |  |  |  |  |  |  |  | x |  |  |  |  |  |  |  |  |  |  |  |
| 97 | *Vaccinium floribundum* | Ericaceae | Upright shrub | X | XX | X | 1600–4600 | 942 |  |  |  | x |  | x | x | x |  |  |  |  | x |  |  |  |  | x |  |
| 98 | *Vaccinium meridionale* | Ericaceae | Upright shrub | XX | XX | X | 1850–3800 | 150 |  |  |  | x | x | x | x | x |  |  | x | x | x | x |  |  | x | x |  |
| 99 | *Valeriana pilosa* | Caprifoliaceae | Basal rosette | - | X | XX | 2650–4800 | 454 |  |  |  |  |  |  |  | x |  |  |  |  |  |  |  |  |  |  |  |
| 100 | *Vallea stipularis* | Elaeocarpaceae | Tree | X | XX | X | 1990–4300 | 900 |  |  |  | x |  |  | x | x |  |  | x | x | x | x | x |  |  | x |  |
| 101 | *Viburnum tinoides* | Adoxaceae | Tree | X | XX | X | 920–3724 | 323 |  |  |  | x |  |  | x | x |  |  |  | x | x |  | x |  |  | x |  |
| 102 | *Weinmannia tomentosa* | Cunoniaceae | Tree | X | XX | X | 1700–3724 | 1508 |  |  |  |  |  |  | x | x |  |  |  | x | x | x | x |  |  | x |  |
| 103 | *Werneria pygmaea* | Asteraceae | Cushion/mat forming | - | X | XX | 3140–4850 | 308 |  |  |  |  |  |  |  | x |  |  |  |  |  |  |  |  |  |  |  |
